# Survival analysis of coagulation disorders: A retrospective study with a 5-year follow-up

**DOI:** 10.1016/j.heliyon.2023.e16376

**Published:** 2023-05-24

**Authors:** Arman Jahangiri, Sara Ahmadi, Hassan Rafieemehr

**Affiliations:** a Department of Emergency Medicine, School of Medicine, Hamadan University of Medical Sciences, Hamadan, Iran; b School of Medicine, Hamadan University of Medical Sciences, Hamadan, Iran; c Department of Medical Laboratory Sciences, School of Paramedicine, Hamadan University of Medical Sciences, Hamadan, Iran

**Keywords:** Survival, Blood coagulation, Bleeding disorders, Hemophilia

## Abstract

**Objective:**

Rare bleeding disorders (RBDs) are the diseases in which patients experience a deficiency of coagulation factors. In the management of these disorders, surveillance is a significant challenge. This study aimed to assess the survival of patients with RBDs in a five-year follow-up.

**Materials and methods:**

This descriptive cross-sectional study was conducted on 146 patients with RBDs who had referred to Be'sat Hospital of Hamadan, Iran from July 2017 to August 2022. A computerized record search was performed to identify the patients. The surveillance time for a five-year follow-up was assessed with the Kaplan–Meier curve. A log-rank test also served to compare the survival rates according to the type of factor.

**Results:**

Out of 146 patients, 117 (80.2%) were males and 29 (19.8%) were females. They were in the range of 2–59 years of age with a mean of 23.11 ± 14.6. The most common disorder was FVIII deficiency (65.8%), and the rarest one was FXIII deficiency (4.8%). The rate of survival for any reason was 54.42 ± 1.3 months. The survival in combined FV and FVIII deficiencies was found to be longer than in the other deficiencies (55.9 ± 5.7), but there was no significant difference (P ≥ 0.05). In contrast, the survival in FXIII deficiency was observed to be lower than the other cases (44 ± 9.6); however, no significant difference was found in this regard (P ≥ 0.05).

**Conclusion:**

The results of this study show that patients with RBDs have different rates of survival, which suggests that identifying high-risk patients may be helpful for the improvement of their survival time through timely therapeutic interventions.

## 1 Introduction

Hemostasis is a process in which the damage to blood vessels is repaired via platelets and coagulation factors [1]. The deficiency of coagulation factors, including fibrinogen or Factor I (FI), prothrombin or FII, FV, FVII, FVIII, FIX, FX, FXI, and FXIII, and combined factor deficiencies such as FV and FVIII are considered as rare bleeding disorders (RBDs) [2]. The worldwide distribution of RBDs has been estimated to be 3–5% by the World Federation of Hemophilia (WFH) [3]. Based on the previous data, FVII deficiency is the most common, while FII deficiency is the rarest bleeding disorder [4]. RBDs are generally categorized as hereditary and acquired diseases. Most RBDs are inherited as autosomal recessive cases. Although homozygous diseases are associated with severe symptoms, heterozygosities have normal factor levels, which make them asymptomatic. Clinical bleeding severity is closely associated with residual coagulant activity [5]. Those suffering from RBDs are at the risk of prolonged bleeding, which can occur inside the vital structures of the body, such as the central nervous system (CNS) or the upper airways. This can be life-threatening [6]. Interestingly, the bleeding tendency increases in the RBD patients who experience coronavirus disease 2019 (COVID-19) [7]. A common way to control coagulation disorders is the injection of coagulation factor concentrates including fresh frozen plasma (FFP) or recombinant products. Patients are at the risk of severe hemorrhage and even death due to the delay in diagnosis or proper treatment. The protocols for prophylactic or replacement treatments with deficient coagulation factors are primary ones whose only goal is to reduce the frequency and intensity of bleeding [8,9]. Patients with RBDs are primarily protected from venous and arterial thrombosis risks. This is done with FFP, which contains all the clotting factors and recombinant concentrates [8].

Despite deploying these supportive treatments, the adverse events of these disorders and surveillance rates are always significant challenges for both patients and healthcare providers. The purpose of this study is to determine the survival time in patients with coagulation disorders. Conducting surveillance for RBDs may be helpful to determine the impacts of the type of coagulation factor deficiency, bleeding, and the development of new treatments.

### 1.1 Materials and methods

This descriptive cross-sectional study was carried out on patients with coagulation disorders who had referred to Be'sat Hospital of Hamadan University of Medical Sciences from July 2017 to August 2022. A computerized record search was conducted to identify all the patients with RBDs. They had to meet specific inclusion and exclusion criteria to be included in the study sample. The inclusion criteria were the diagnosis of RBDs, complete information recorded in the medical records, and death-causing bleeding disorders. The patients were excluded from enrollment in the study if they had incomplete information in the medical records, had died due to other blood disorders (aplastic anemia, hemolytic anemia, leukemia, and thrombocytopenia), or refused to enroll in this study. Based on the definition proposed by the Scientific and Standardization Committee (SSC) of the International Society on Thrombosis and Hemostasis (ISTH) [10], in the current study, the deficiency of FVIII and FIX was defined as severe if the baseline factor activity level was less than 1%, moderate if from 1% to 5%, and mild if > 5–40% of normal. For the patients who had died, the supplemental information was obtained from their families. Informed consent was obtained from each subject or the parents of minor children before the data collection. Also, approved informed consent was taken from the families of the dead patients. The survival time for each patient was calculated from the first year when RBD was diagnosed. This study was approved by the ethics committee of Hamadan University of Medical Sciences (IR.UMSHA.REC.1401.386; No. 140105043325).

### 1.2 Statistical analysis

Descriptive statistical methods including the use of frequencies, percentages means and standard deviation (SD) were implemented to analyze the demographic and other quantitative variables. The survival time in a five-year follow-up program was assessed with the Kaplan–Meier method. The log-rank test was also used to compare the rates of survival according to the type of factors. All the statistical analyses were carried out using SPSS version 22. The *P-*value of <0.05 was considered statistically significant.

## 2 Results

**Fig. 1:**
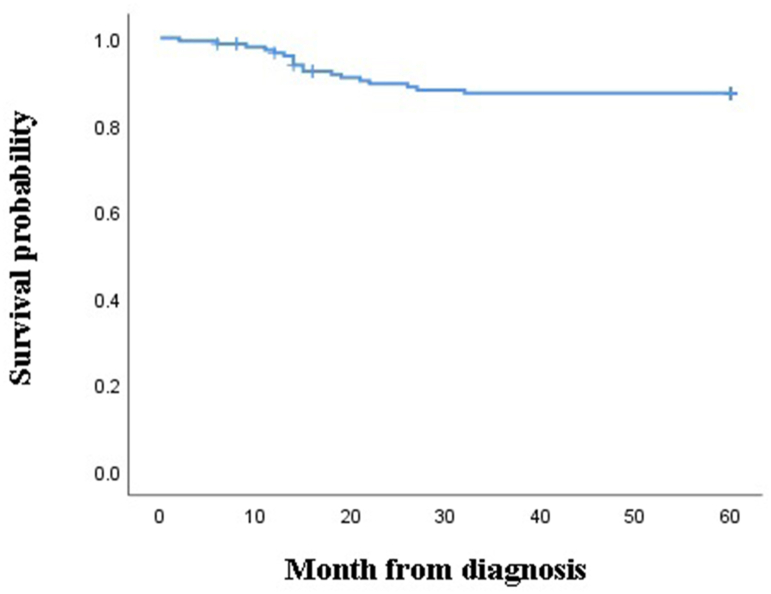
The 5-year survival of patients with coagulation disorders.

**Fig. 2:**
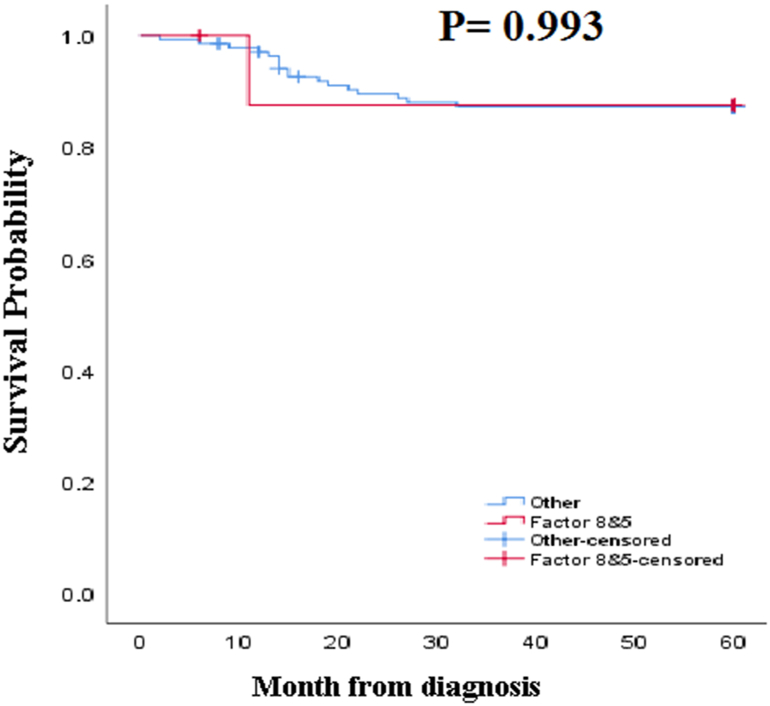
The 5-year survival of patients with combined FV and FVIII deficiency in comparison with other RBDs: The average survival of patients with combined FV and FVIII deficiency was 55.9 months. For the other RBD cases, it was 54.3 months. Based on the results of the log-rank test, there was no statistically significant difference of survival time between the two groups of patients (P = 0.993).

**Fig. 3:**
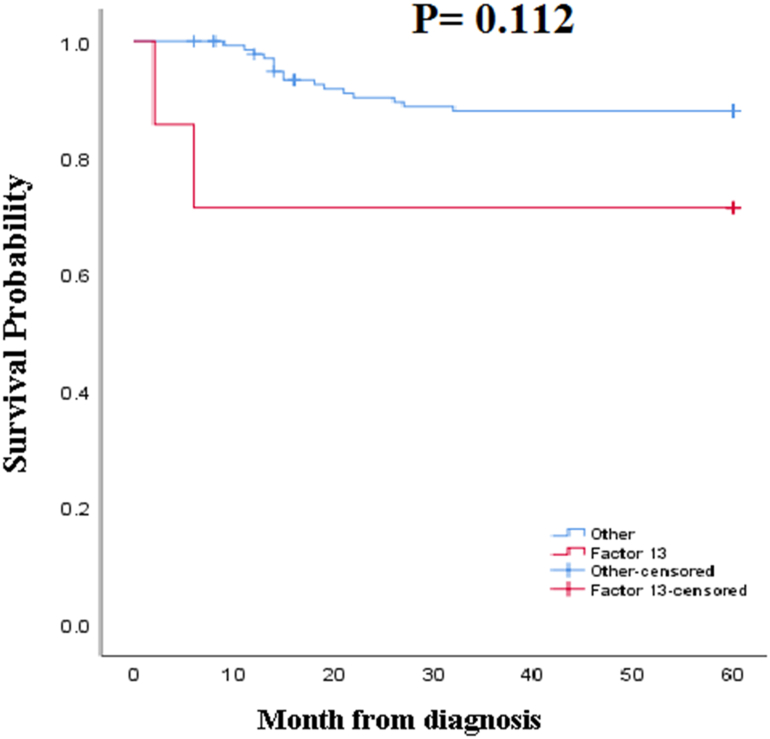
The 5-year survival of patients with FXIII deficiency in comparison with the other RBD patients: The average survival of patients with FXIII deficiency was 44 ± 9.6 months. For the other RBD cases, it was 54.9 ± 1.2 months. Based on the results of the log-rank test, there was no statistically significant difference of survival time between the two groups of patients (P = 0.112). (+) indicates FXIII censoring.

Based on the inclusion and exclusion criteria, 146 patients with RBDs, including deficiencies of FVIII, FIX, FV, FX, FXIII, and combined FV and FVIII, provided the data required for the analyses. The baseline demographic data are presented in Table 1. There were 117 (80.2%) males and 29 (19.8%) females with a mean age of 23.11 ± 14.6 years in the range of 2–59. As shown in Table 1, most of the patients were in the age group of 30–40 and less than ten years old. A positive family history of RBDs was reported by 91 (62.3%) of the subjects. Out of them, 85 (91.3%) patients had a family history of FVIII deficiency. In this study, the most common disorder was FVIII deficiency (65.8%), and the rarest disorder was FXIII deficiency (4.8%) (Table 1). Using the survival time assay in a 5-year follow-up, the patients' survival for any reason was found to be 54.42 ± 1.3 months (Fig. 1). The survival in the case of combined FV and FVIII deficiency was higher than that in the other cases of deficiency (55.9 ± 5.7), but no significant difference was found (Fig. 2) (P = 0.993). Also, the survival of the patients with FXIII deficiency was lower than the others (44 ± 9.6), but no significant difference was found (Fig. 3) (P = 0.112). It should be noted that the average survival time of the patients with FXIII deficiency was lower than that of all the other patients (see Table 2).

**Table 1:** Demographic and clinical characteristics of patients.

Characteristics'	Patients (n = 146)
Age (years)	
Mean + SD	23.11 ± 16.4
Range	(2–59)
≤10	34 (23.3%)
10–20	32 (22%)
20–30	23 (15.7%)
30–40	39 (26.7%)
≥40	18 (12.3%)
Sex; n(%)	
Male	117 (80.2%)
Female	29 (19.8%)
Factor deficiency; n(%)	
FVIII	96 (65.8%)
Severe	56 (58.3%)
Moderate	17 (17.7%)
Mild	23 (24%)
FIX	10 (6.8%)
Severe	2 (20%)
Moderate	5 (50%)
Mild	3 (30%)
FV	16 (11%)
FX	8 (5.5%)
FV& VIII	9 (6.2%)
FXIII	7 (4.8%)
Family history of RBDs, n(%)	
Yes	91 (62.3%)
No	55 (47.7%)

**Abbreviations: F:** Factor; **RBDs:** Rare bleeding disorders; **N:** Number; **SD:** Standard deviation.

**Table 2:** Comparison of survival time between coagulation factors deficiency.

Factors deficiency	Mean survival time (Months, Mean + SD)	Mean survival time of Other deficiency (Months, Mean + SD)	*P*-value
FVIII	55.5 ± 1.3	52.3 ± 2.5	0.313
FIX	51.5 ± 3.4	54.6 ± 1.3	0.483
FV	54.5 ± 3.7	54.4 ± 1.3	0.982
FX	54.2 ± 5.2	54.4 ± 1.3	0.988
FV & VIII	55.9 ± 5.7	54.3 ± 1.2	0.993
FXIII	44 ± 9.6	54.9 ± 1.2	0.112

**Abbreviations: F:** Factor; **SD:** Standard deviation.

## 3 Discussion

Persons with RBDs have deficient coagulation factors that are necessary for normal hemostasis [1]. Since most RBDs are inherited as autosomal recessive cases, a diverse range of minimal to life-threatening clinical symptoms has been observed in patients. Hemophilia is an X-linked inherited and common disorder in which patients experience spontaneous or internal bleeding symptoms. Based on the data from previous studies, soft tissue and joint bleeding are common in severe FI, FII, FVIII, and FX deficiencies. However, patients with FXIII deficiency tend to have umbilical cord and CNS bleeding coagulation [11,12]. The association between the severity of clinical symptoms of RBDs and survival rate also varies. Survival rate may be affected by several factors, such as burdensome and timely disease diagnosis, the severity of symptoms, and treatment [[13], [14], [15]].

In this study, the survival time of RBDs patients was evaluated in a 5-year follow-up program. Most of the subjects were under thirty years of age. This is comparable with the findings of Soucie et al. [16], who showed that the mean age of hemophilia is less than 35 years. The younger overall age distribution of the patients in the current study implies the importance of early diagnosis and clinical management of RBDs. Of the patients, 117 (80.2%) were males who suffered from FVIII deficiency, which is a sex-related chromosomal disorder. These findings are based on the fact that hemophilia A is a male disorder [17]. In this regard, awareness of male infants or even male embryos through screening, especially in families with a history of hemophilia, is crucial. The assessment of survival in RBD patients was the primary purpose of this study. In this regard, the data gained in the follow-up surveillance showed that the average survival was 54.42 ± 1.3 months for all the patients with various bleeding disorders. So far, very few studies have investigated the survival time in RBDs. One example is Ernstbrunner et al. [18]. As they found, the survival for any reason was 78% and 59% in 15 and 20 years of follow-up, respectively [18]. Since the causes of death in RBDs are not unique and often co-occur, the regular evaluation of clinical and laboratory findings can to help manage the disease so as to increase the survival time of patients. Despite the small number of the patients with FXIII deficiency in this study, their survival time was lower than the others although the difference was not significant. This finding generally corresponds to the fact that the bleeding symptoms of FXIII typically occur soon after birth from the umbilical stump and are associated with low survivance of patients [19]. In addition, intracranial hemorrhage is a life-threatening symptom of FXIII deficiency that significantly impacts the survival time [20,21]. It is to be noted that, in this study, CNS bleeding was the common cause of death in patients with FXIII deficiency. This suggests the importance of optimal monitoring and timely treatment to manage severe bleeding in patients with FXIII deficiency.

In contrast to FXIII deficiency, the longest survival was observed in patients with FV and FVIII deficiency, but no significant difference was found between them and the other RBD cases. This finding suggests that combined FV and FVIII deficiency is associated with moderately reduced FV and FVIII as well as mild bleeding symptoms [22]. Regular treatment with FFP, which contains enough FV and FVIII, can be helpful in the management of patients with FV and FVIII deficiency so as to give them longer survival.

## 4 Conclusion

In summary, it was found that FVIII deficiency is a common RBD, especially in the male gender. In addition, a family history of FVIII deficiency is a potential risk factor for RBD incidence. Although FXIII deficiency is a rare bleeding disorder, those afflicted with it have a shorter survival than other RBD cases. Since RBDs patients have different rates of survival, identifying high-risk patients and choosing an appropriate treatment strategy may be helpful for the improvement of their survival. This descriptive cross-sectional research has some limitations. Firstly, there are numerous risk factors associated with RBDs survival, but many of them have been ignored in this study. Secondly, due to the limited time span of the study, the patients were not followed up after five years. Thirdly, the effect of treatment on the occurrence of bleeding complications or the mortality rate of the patients was not investigated. Accordingly, further studies with a larger number of patients and a longer time interval are needed to accurately determine the survival time of patients by evaluating the factors involved in the prognosis of the disorders.

### Author contribution statement

Arman Jahangiri: Conceived and designed the experiments, contributed reagents, materials, analysis tools and data.

Sara Ahmadi: Performed the experiments, wrote the paper.

Hassan Rafieemehr: Conceived and designed the experiments, performed the experiments, analyzed and interpreted the data, contributed reagents, materials, analysis tools and data; wrote the paper.

## Data availability statement

Data will be made available on request.

## Additional information

No additional information is available for this paper.

## Declaration of competing interest

The authors have no interests to declare.

## References

[1] O'donnell J.S., O'sullivan J.M., Preston R.J. (2019). Advances in understanding the molecular mechanisms that maintain normal haemostasis. Br. J. Haematol..

[2] Palla R., Peyvandi F., Shapiro A.D. (2015). Rare bleeding disorders: diagnosis and treatment. Blood.

[3] Jain S., Acharya S.S. (2020). Rare coagulation factor deficiencies. Hem. Adol. Fem..

[4] Batsuli G., Kouides P. (2021). Rare coagulation factor deficiencies (Factors VII, X, V, and II). Hematol./Oncol. Clin..

[5] Menegatti M., Peyvandi F. (2019). Treatment of rare factor deficiencies other than hemophilia. Blood.

[6] Peyvandi F., Menegatti M., Palla R. (2013). Rare bleeding disorders: worldwide efforts for classification, diagnosis, and management. Semin. Thromb. Hemost..

[7] Dorgalaleh A., Dabbagh A., Tabibian S., Bahraini M., Rafieemehr H. (2021). Persistent hiccups in a patient with mild congenital factor V deficiency and COVID-19; clinical and laboratory finding of a rare bleeding disorder. Int. J. Lab. Hematol..

[8] Shapiro A. (2020). The use of prophylaxis in the treatment of rare bleeding disorders. Thromb. Res..

[9] Peyvandi F., Garagiola I., Biguzzi E. (2016). Advances in the treatment of bleeding disorders. J. Thromb. Haemostasis.

[10] Blanchette V.S., Srivastava A. (2015). Definitions in hemophilia: resolved and unresolved issues. Semin. Thromb. Hemost..

[11] Jain S., Acharya S.S. (2018). Management of rare coagulation disorders in 2018. Transfus. Apher. Sci..

[12] Rafieemehr H., Dorgalaleh A., Mansouritorghabeh H. (2020). Mining of mortality-related findings in rare bleeding disorders: a retrospective study from two centers. Blood Res..

[13] Lassila R., Makris M. (2016). Safety surveillance in haemophilia and allied disorders. J. Int. Med..

[14] Soucie J.M., Miller C.H., Kelly F.M., Payne A.B., Creary M., Bockenstedt P.L., Kempton C.L., Manco-Johnson M.J., Neff A.T., Investigators H.I.R.S. (2014). A study of prospective surveillance for inhibitors among persons with haemophilia in the United States. Haemophilia.

[15] Byams V., Kouides P., Kulkarni R., Baker J., Brown D., Gill J., Grant A., James A., Konkle B., Maahs J. (2011). Surveillance of female patients with inherited bleeding disorders in United States Haemophilia Treatment Centres. Haemophilia.

[16] Soucie J.M., Miller C.H., Dupervil B., Le B., Buckner T.W. (2020). Occurrence rates of haemophilia among males in the United States based on surveillance conducted in specialized haemophilia treatment centres. Haemophilia.

[17] Konkle B.A., Fletcher S.N. (2022). Hemophilia A. GeneReviews. NIH.

[18] Ernstbrunner L., Hingsammer A., Catanzaro S., Sutter R., Brand B., Wieser K., Fucentese S.F. (2017). Long-term results of total knee arthroplasty in haemophilic patients: an 18-year follow-up. Knee Surg. Sports Traumatol. Arthrosc..

[19] Muniraman H., Sardesai T., Sardesai S. (2018). Disorders of the umbilical cord. Pediatr. Rev..

[20] Alavi S.E.R., Jalalvand M., Assadollahi V., Tabibian S., Dorgalaleh A. (2018). Intracranial hemorrhage: a devastating outcome of congenital bleeding disorders—prevalence, diagnosis, and management, with a special focus on congenital factor XIII deficiency. Semin. Thromb. Hemost..

[21] Naderi M., Zarei T., Haghpanah S., Eshghi P., Miri-Moghaddam E., Karimi M. (2014). Intracranial hemorrhage pattern in the patients with factor XIII deficiency. Ann. Hematol..

[22] Shao Y., Wu W., Xu G., Wang X., Ding Q. (2019). Low factor V level ameliorates bleeding diathesis in patients with combined deficiency of factor V and factor VIII. Blood.

